# Automatic Microaneurysms Detection for Early Diagnosis of Diabetic Retinopathy Using Improved Discrete Particle Swarm Optimization

**DOI:** 10.3390/jpm12020317

**Published:** 2022-02-20

**Authors:** Usharani Bhimavarapu, Gopi Battineni

**Affiliations:** 1 Department of Computer Science and Engineering, Koneru Lakshmaiah Education Foundation, Vaddeswaram 522502, Andhra Pradesh, India; ushafdp1122@gmail.com; 2 Clinical Research Center, School of Medicinal and Health Products Sciences, University of Camerino, 62032 Camerino, Italy

**Keywords:** diabetic retinopathy, fuzzy image processing, microaneurysms, PSO clustering, swarm intelligence

## Abstract

Diabetic retinopathy (DR) is one of the most important microvascular complications associated with diabetes mellitus. The early signs of DR are microaneurysms, which can lead to complete vision loss. The detection of DR at an early stage can help to avoid non-reversible blindness. To do this, we incorporated fuzzy logic techniques into digital image processing to conduct effective detection. The digital fundus images were segmented using particle swarm optimization to identify microaneurysms. The particle swarm optimization clustering combined the membership functions by grouping the high similarity data into clusters. Model testing was conducted on the publicly available dataset called DIARETDB0, and image segmentation was done by probability-based (PBPSO) clustering algorithms. Different fuzzy models were applied and the outcomes were compared with our probability discrete particle swarm optimization algorithm. The results revealed that the proposed PSO algorithm achieved an accuracy of 99.9% in the early detection of DR.

## 1. Introduction

Diabetic retinopathy (DR) is one of the complications of diabetes that affects eyesight and leads to complete vision loss [1]. It damages the blood vessels of the tissues at the back of the retina. Reports state that one in ten people is diagnosed with diabetes, and across the world, 420 million people had diabetes in 2020; this is expected to double by 2045. Diabetes severely affects different body organs, including the eyes (i.e., DR), kidneys (i.e., diabetic nephropathy), and nerves (i.e., diabetic neuropathy) [2,3,4]. The heart failure risk in diabetic patients is greater than in non-diabetic patients, and 19–30% of diabetic patients are prone to the risk of heart failure [5,6,7]. The risk of heart failure due to Type 1 and Type 2 diabetes are over 30% and 10%, respectively, which is higher than the risk caused by smoking and other coronary diseases [8]. Additionally, this disease can damage the kidneys, and diabetic nephropathy occurs in 20 to 40% of diabetic patients [9].

The number of people diagnosed with DR is growing exponentially across the world. Two phases are defined: non-proliferative and proliferative [10]. In non-proliferative DR, the different signs are microaneurysms, haemorrhages, exudates (hard and soft), cotton wool spots, and inter-retinal microvascular abnormalities. Proliferative DR is an advanced stage of diabetic retinopathy. In this stage, new abnormal blood vessels grow in different regions of the retina and cause total blindness. Microaneurysms are the first symptom in DR detection. 

Computers can now learn from large datasets in many areas by using deep learning algorithms. In medical imaging, including retinal images, several deep learning algorithms for the classification or detection of certain disease conditions have been developed. Early literature discusses the employment of fuzzy logic in the identification of DR and diagnosis of its gravity. In [11], support vector machines coupled with fuzzy logic (Fuzzy SVM) were applied to identify DR, achieving 89.02% accuracy [11]. Using fuzzy morphological techniques, DR diagnosis can be achieved with an accuracy of 93.8% [12]. Similarly, Almotiri et al. applied fuzzy c-means (FCM) clustering for image segmentation, and DR detection was achieved with 95.88% accuracy [13]. 

It is reported that by adopting the segmentation, resize, contrast stretching, opening, and closing operations with the fuzzy classifier logic, the accuracy improves to 95.63% [14]. A diabetic classification model for the detection of levels of DR severity using the particle swarm optimization (PSO) with convolution neural network algorithm was done in [15]. These advanced techniques, such as HSI image, median filter, CLAHE, and applied segmentation techniques, can reach a maximum value of 99.84% accuracy in early DR detection [16]. However, the scarcity of research on detecting DR to reduce the risk of vision loss using advanced fuzzy techniques motivated the present study’s conduction. 

We considered a publicly available dataset called DIARETDB0 (of 130 images) with different DR severity levels, such as no DR, mild, moderate, severe, and proliferative DR. This work aimed to propose a novel fuzzy algorithm with probability discrete particle swarm optimization (PDPSO) to detect microaneurysms. In addition, pre-processing to progress the image disparity by applying fuzzy image enhancement techniques was performed, and a discrete PSO clustering algorithm was used for segmentation. Further, the performance of the proposed model was compared with existing fuzzy algorithms. 

## 2. Materials and Methods

### 2.1. DIARETDB0 and Images

In this paper, the fundus images were pre-processed by excluding noise from the fundus image, which was further processed using enhancement techniques to highlight the details of the image. Fundus images are two-dimensional images of three-dimensional retina tissue, and various retina structures can be better visualized [17]. Fundus images provide a sharp, high-contrast image of the retina within the focal plane. Table 1 presents the severity levels of diabetic retinopathy. 

**Figure 1:**
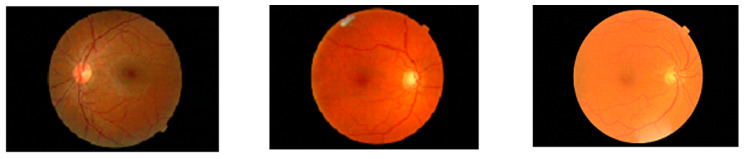
No DR (**left**), microaneurysms (**middle**), and hemorrhages (**right**) [19].

**Figure 2:**
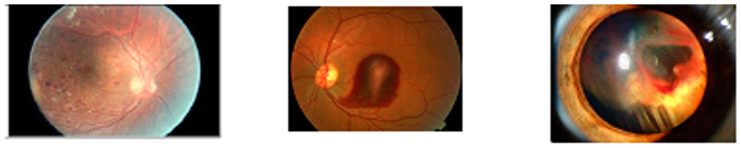
Neovascularisation (**left**), preretinal hemorrhage (**middle**), and retinal detachment (**right**) [20,21,22].

In color fundus photography, one can identify three types of hemorrhages, called dot-blot, flame-shaped, and boat-shaped hemorrhages. Retinal hemorrhages bleed blood in the retina of the eye due to blood accumulating in the outer or inner nuclei. Exudates are bright, reflective, white, or cream-colored lesions that appear on the retina. Generally, the pixel values of the exudates are brighter than the other region pixels. Figure 1 and Figure 2 show the early stages of non-proliferative and proliferative DR. 

### 2.2. Experimental Framework

Neuro-fuzzy models are used in medical research, especially in diabetes. Neuro-fuzzy refers to the grouping of artificial neural networks (ANN) and fuzzy logic (FL). The main advantages of fuzzy image processing are that it:

- Processes the medical image in the pixel-by-pixel form.
- Overcomes the difficulties in the greyness ambiguity, geometrical fuzziness, and complex features of the images.
- Deals with the uncertain information in the images.
- Provides fast computation using fuzzy operations. The present discrete PSO clustering variants model is applied to the publicly available diabetic retinopathy datasets in DIARETDB0.

**Figure 3:**
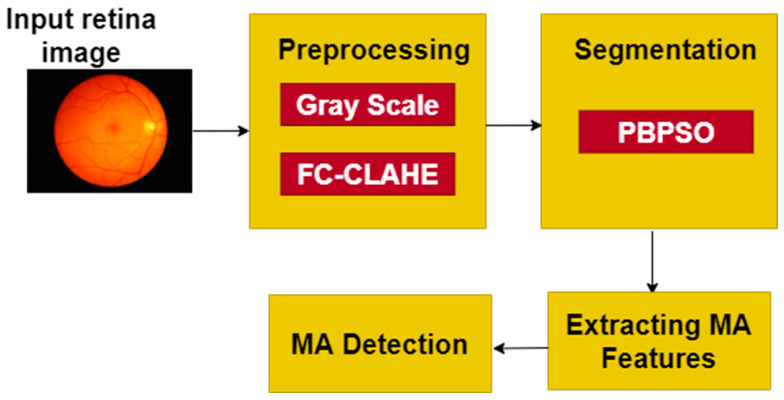
A proposed experimental framework.

The new probability discrete PSO clustering algorithms give higher accuracy than the other swarm intelligence algorithms. Figure 3 represents the experimental framework for the model developed in the detection of aneurysms by using swarm intelligence techniques. 

### 2.3. Neuro-Fuzzy Pre-Processing

In medical science, the available data are in raw format, which is relatively hard to understand without preprocessing, and the disease severity is expressed in linguistic terms as low, medium, and high [23]. Image enhancement plays a crucial part in retinal image examination and processing. Conventional contrast enhancement algorithms like histogram equalization (HE) [24] or contrast limited adaptive histogram equalization (CLAHE) are not suitable for retinal images due to the variation of the dynamic density and amplification of noise. 

#### 2.3.1. Fuzzy Contrast Enhancement 

**Figure 4:**
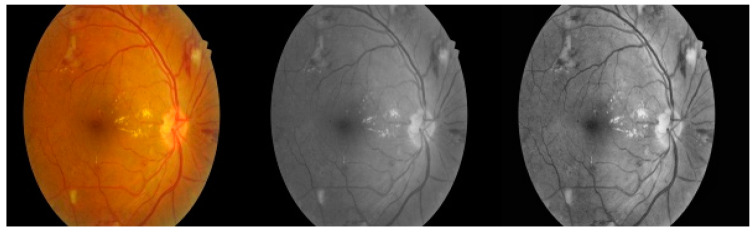
Resized image (**left**), grey image (**middle**), and after applying FC-CLAHE (**right**).

To improve the image contrast, one of the standard techniques used is HE [24]. Fuzzy image enhancement is the technique to produce an image of more sophisticated divergence than the original image. High-resolution fundus images are hard to interpret, so pre-processing of the image is required to improve the image’s excellence. The main advantage of pre-processing is to classify the input image effectively. We applied fuzzy image processing to convert the color image to the grayscale image and to proceed further. For better visualization and detection of the red lesion for medical images, a technique called FC-CLAHE [25] was used, and the fuzzy logic contrast enhancement technique helped to improve the retina image in the proposed model. The pre-processing phase of the retina image is shown in Figure 4. 

#### 2.3.2. Image Segmentation 

**Figure 5:**
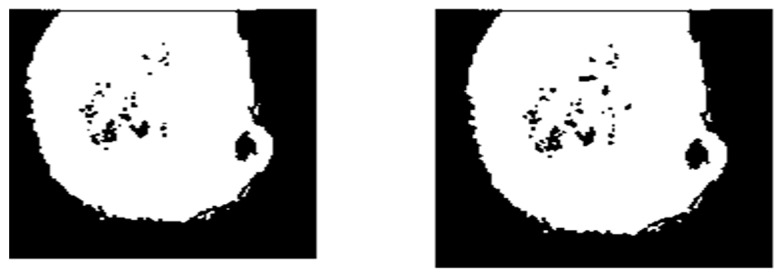
PSO clustering (**left**), and PBPSO clustering (**right**).

The primary objective of the segmentation is to reorganize or modify the retina image’s appearance to make it simpler to differentiate and extract the features of the retina image. For improved segmentation and to improve the accuracy of the segmented image, optimization algorithms are employed. In this work, discrete particle swarm optimization clustering was applied to segment the pre-processed image. The segmentation phase improved the detection of the microaneurysms from the retina image. In this paper, we used the probability-based particle swarm optimization (PBPSO) clustering algorithms to segment the pre-processed image (Ref Figure 5). 

#### 2.3.3. PBPSO Algorithm

In PBPSO, the clustering algorithm initializes all particles in the swarm to measure the fitness of each particle via a discrete search heuristic (DSH). Depending on fitness, each of the primary particles calculates the objective function and velocity in the cluster block. This procedure is applied to each particle. To provide the initial population of the particles, the three steps involved are to:

- Generate the k-random numbers within the range (1, N). Group these data points into different k cluster blocks.
- Group the remaining N−K data points into different k clusters.
- Randomly append the cluster separator between the clusters to generate the particle sequence.

The velocity generation procedure of the particles is used to identify the clusters in all the current and the G-best particles. Thereafter, construction of the cluster velocity and the particle sequence is done, and each particle constructs the new sequence based on the changed probabilities from the velocities. For the generated clusters, the normalized probabilities are calculated to select the clusters to construct the new particles. By using the probability distribution range for the corresponding random numbers, clusters are selected. The final particles are generated from the selected clusters by using the random pseudo sequence. Building the pseudo sequence by appending the selected cluster to the already existing pseudo sequence is then done to check the data points in the new cluster. The data points are eliminated if they occur a second time. For the centroid updating, every particle updates the newly constructed sequence using the centroid updating phase for improving the sequence fitness. The fitness measure is the primary accuracy of the segmented retina image. For each image pixel in this study, we evaluated the fitness measures to quantify the quality of the clusters, and irrelevant or redundant features were removed from the segmented image to enhance accuracy. All of these experiments were conducted in a PyTorch on the Intel Core i5 3.4 GHz processor for experimentation. 

### 2.4. Fitness Measures

The fitness measure is the primary performance of the segmented retina image. For each pixel of the image, the fitness measure in the retina image is evaluated. The fitness measures discussed in this paper are the measures to quantify the quality of the clusters. The fitness measures used for the proposed PSO algorithm are:

$$\mathrm{fuzzy}\;\mathrm{entropy}:\;\sum_{i}\sum_{j}\frac{y_{\mathrm{ij}}}{\log y_{\mathrm{ij}}}$$

$$\mathrm{kurtosis}:\;\frac{1/n\sum {\left(y-\bar{y}\right)}^{2\;}}{s^{4}}$$

$$\mathrm{skewness}:\;\frac{1/n\sum {\left(y-\bar{y}\right)}^{3\;}}{s^{3}}$$

$$\mathrm{correlation}:\;\frac{\sum_{i}\sum_{j}\left(\mathrm{ij}\right)y_{\mathrm{ij}}-\mu_{i}\mu_{j}}{s_{i}s_{j}}$$

$$\mathrm{variance}\;\frac{\sum_{i=1}^{N}{\left(y_{i}-\bar{y}\right)}^{2}}{N-1}$$

where y is the yth dimension for (i, j) and µ, $\bar{y}$ is the mean, and S is the standard deviation. N is the total number of samples. The size of the retina image is MXN. Fuzzy entropy achieves efficient retina image segmentation and improves the convergence speed, thus improving the robustness of the retina image. It calculates the similarity values for each feature. Kurtosis determines the normality of the data distribution and confirms the symmetry of the retina image. The skewness signifies the asymmetry of the cluster, whereas the kurtosis embodies the concentration of the cluster in the retina images. In digital image processing, the kurtosis values are interpreted with noise, i.e., high kurtosis values mean low noise and good resolution in the image. The variance of the image is how the pixels spread in the image; high variance means the resolution and the contrast of the image.

### 2.5. Feature Extraction

Grey level co-occurrence matrix (GLCM) functions characterize the texture of the retina image and extract the measures from this matrix. The relative frequency of the pixels in the retina image is defined in the matrix dimension. The measures determined by the means of the features are homogeneity, dissimilarity, energy, contrast, and mean.

The features for the detection of the microaneurysm are:

$$\mathrm{homogeneity}=\sum_{i}\sum_{J}\frac{1}{1+{\left(1-j\right)}^{2}}P_{\mathrm{ij}}$$

max. probability = max{p_ij_(i, j)}

energy = ∑∑P_ij_^2^

dissimilarity = ∑∑|i − j| p_ij_

$$\mathrm{contrast}=\sum_{i=0}^{\infty }\sum_{j=0}^{\infty }P(i-j{)}^{2}$$

$$\mathrm{mean}=\sum_{i}\sum_{j}\frac{P_{\mathrm{ij}}}{N*M}$$

In this, p is the probability of (i, j) at various distances, and N, M are the dimensions of the image.

## 3. Results 

The image dataset was decomposed into two sets for training (80%) and testing (20%) purposes. Therefore, 104 images were utilized for model training, and the remaining 26 were used for testing. The performance measures on the fuzzy enhanced image are tabulated in Table 2.

The main challenge in image clustering is how to employ clusters based on the earlier complex interpretations and to find the new cluster. The remaining cluster algorithms used the mean, median, variance, and covariance to characterize the cluster. Finding new clusters and combining the old and new clusters was problematic. To overcome this, new clusters were merged with old clusters by examining the entropy, kurtosis, and skewness statistics. If the two clusters had either unequal kurtosis or unequal skewness of the data, both clusters were tested for normality. If there was normality, two clusters were merged despite the inequality of their kurtosis and skewness. Table 3 presents the performance measures on the fuzzy enhanced image. A comparison of the different metrics, i.e., sensitivity, specificity, and accuracy on different severity levels of diabetic retinopathy, is presented in Table 4. 

Table 4 presents the evaluation of the performance metrics for the improved PSO. The table shows that the proposed model achieved a high percentage of accuracy. According to the results, the overall testing accuracy and the performance metrics in the improved PSO are appropriate for the detection of microaneurysms, demonstrating a testing accuracy of 99.9%. The proposed algorithm achieved an accuracy of 99.99%, which is greater than the existing techniques. The proposed algorithm also achieved 99.8% and 99.1% for sensitivity and specificity, respectively. 

## 4. Discussion

To predict the medical diagnosis of diabetes, the proposed improved discrete particle swarm optimization can be used as a global approach. Between 10 and 30% of diabetic patients can develop DR [29,30,31,32], but the early detection of microaneurysms can reduce the risk severity of DR [33]. Thus, it can be suggested that treatment may be possible by improving the diet, exercising, and carefully monitoring blood sugar levels.

Some authors used pre-processing techniques such as normalization, class balancing, and feature selection with the classifier ANFIS (correlation coefficient, PCA) using the DIARETDB0 and local datasets, and obtained the accuracy of 84.09% and 65.90%, respectively [34]. Other pre-processing techniques used were color normalization, median filter, histogram specification, and grayscale with the classifier ANFIS using local datasets, which obtained 98.55% accuracy and sensitivity [35]. It is also reported that pre-processing techniques including light equalization, vessel enhancement, adaptive binary thresholding, and lesion removal with the classifier Mamdani fuzzy logic controller can achieve maximum classification accuracy [36].

Rahim et al. [37] used the fuzzy histogram equalization pre-processing approach and applied the segmentation techniques of morphological operations, FRANGI filter, kirsch filter, entropic thresholding with the classifier KNN, polynomial support vector machine kernel, RBF kernel support vector machine, and NB, and obtained an accuracy of 93%. The segmentation techniques of morphological operations, edge detection, and circular hough transform were used with the classifier fuzzy support vector machines [38] to tune the multi-layer perceptron neural network weights to classify diabetes mellitus and its types [39]. Beschi et al. [40] proposed a novel methodology to predict Type 2 diabetes using particle swarm optimization and the fuzzy means clustering algorithms and obtained an accuracy of 95.42% on the PIDD dataset.

In this paper, the image was resized to 250 × 250 pixels at the pre-processing stage. The resized image was then subjected to contrast enhancement by utilizing the Fuzzy Clipped Contrast-Limited Adaptive Histogram Equalization (FCCLAHE) algorithm to identify the lesions in the image. In the next step, we performed segmentation to obtain useful lesion information from the image, followed by feature extraction, in differentiating the candidate regions as microaneurysms or non-microaneurysms. Thus, our study produced a mean accuracy of the retinal multi-image classification of 99.99%. The results demonstrate how accurate the proposed discrete PSO clustering model is in the detection and classification of diabetic retinopathy using the publicly available dataset (DIARETDB0), achieving an accuracy, sensitivity, and specificity of 99.99%, 99.8%, and 99.1%, respectively. 

The shortcomings of the existing studies mean that, so far, they have failed to reduce the execution time, high computational costs, and computational complexities associated with retinopathy classification. We proposed the discrete PSO clustering algorithm to tackle these problems, which reduces the execution time. The proposed system uses fuzzy pre-processing techniques, which deal with greater robustness to contrast and manage vagueness efficiently, reducing the computational complexities.

## 5. Conclusions

This paper proposed a novel approach to microaneurysm detection using probability particle swarm optimization. Fuzzy image enhancement was initially carried out due to its ability to effectively handle the uncertainty, ambiguity, and vagueness of the image data. The core advantage of the fuzzy technique lies in the usage of the membership functions that enhance the model’s ability to handle image ambiguity. By applying our approach, the image segmentation and feature extraction ultimately helped to classify the lesion type. The experimental results demonstrate that the proposed approach classifies microaneurysms with 99.9% accuracy.

## Figures and Tables

**Table 1:** Diabetic retinopathy (DR) severity levels [18].

Severity Level	Description
No NPDR	No abnormalities
Mild NPDR	Microaneurysms only
Moderate NPDR	More microaneurysms and exudates
Severe NPDR	Intraretinal microvascular anomalies, intraretinal hemorrhage, abnormal blood vessel growth
PDR	Neovascularization, preretinal hemorrhage
Gestational DR	Pregnancy itself is a risk factor for the progression of DR

**Table 2:** Performance measures on the fuzzy enhanced image.

Metric	Average
Mean	−3.54009 × 10^−4^
Variance	12.3746
Lambda	0.12403
Correlation	−3.54009161
Kurtosis	0.7734
Skewness	1.5332

**Table 3:** Similarity measures on the segmented image.

ID	PSO				Discrete PSO			
	Entropy	Kurtosis	Skew	Runtime	Entropy	Kurtosis	Skew	Runtime
Image01	3.57	−0.75	−1.11	16.45	2.58	−0.83	−1.25	16.34
Image02	4.33	−1.8	0.39	19.51	4.38	−1.9	0.28	18.89
Image03	4.10	−1.94	−9.25	19.47	4.23	−1.98	−9.53	18.98
Image04	4.91	−0.87	1.06	20.64	4.99	−0.97	0.99	19.29
Image05	4.20	−1.82	0.41	18.28	4.45	−1.93	0.33	17.85
Image06	1.24	−1.46	−0.73	17.88	1.34	−1.23	0.87	16.67
Image07	4.10	−1.85	−0.38	18.19	4.28	−1.94	−0.45	17.76
Image08	3.57	−1.89	−0.34	18.30	3.87	−1.94	−0.41	17.89
Image09	4.07	−1.78	0.47	18.85	4.89	−1.11	0.59	17.85
Image10	4.78	−0.45	1.25	18.14	4.97	−0.51	1.33	17.56
Image11	4.23	−1.79	−0.46	18.25	4.51	−1.99	−0.89	17.56
Image12	3.25	0.94	−1.72	18.41	3.48	0.89	−1.87	17.85
Image13	4.52	6.13	5.28	18.08	4.89	2.23	2.87	17.67
Image14	4.01	−1.99	−0.07	18.25	3.34	0.97	0.98	16.49
Image15	4.23	−1.94	0.24	18.24	3.46	0.80	0.15	17.51
Image16	4.23	3.07	2.25	18.01	3.45	2.87	1.73	17.65
Image17	4.18	−0.89	1.05	18.53	4.08	0.96	1.03	17.45
Image18	3.15	0.73	−1.65	18.03	3.12	0.71	−1.78	17.88
Image19	4.16	−1.99	−0.10	18.08	4.08	0.98	0.89	17.68
Image20	4.28	−1.99	0.01	18.33	4.15	0.87	0.01	17.83

**Table 4:** Performance measure comparison.

Algorithm	Accuracy (%)	Sensitivity (%)	Specificity (%)
PSO-GIT2FMS [26]	98.4	96	96
DeepCNN [27]	99.1	98.4	97.1
MSRnet [28]	97	82.0	98.3
Proposed	99.99	99.8	99.1

## Data Availability

Not applicable.
